# A transit map for micro-scale urban development in Alexandria, Egypt

**DOI:** 10.12688/f1000research.125816.2

**Published:** 2023-05-09

**Authors:** Mohamed H. Seoudy, Adel El Menshawy, Amr El Adawy

**Affiliations:** 1 Department of Architectural, Faculty of Fine Arts, Alexandria University, Alexandria, Egypt; 2 Department of Architectural Engineering & Environmental Design, Arab Academy for Science Technology & Maritime Transport, Alexandria, Egypt

**Keywords:** Micro-Scale, Transport, Built Environment, Transit-Oriented Community, Transport Supply System, Sustainable Development, Mobility Hub

## Abstract

**Background:** Due to Egypt's strategic location among countries, transportation is one of the most significant development sectors because it plays a major part in today's economy and society and has a large influence on growth and employment. Over the years, the Egyptian General Organisation of Physical Planning (GOPP) has prepared strategic general urban plans in collaboration with local and foreign organisations, including transportation plans. The constant focus of authorities on strategic plans and their inability to implement them on schedule are a major issue. In other words, they always take development from a distant perspective and do not deal with the main problem that exists within cities, as the existing micro-scale transit built environments (MSTBEs) of cities are not ready due to a lack of transit-oriented communities (TOCs), sustainably developed transit supply systems, and mobility hubs.

**Methods: **The "Enhanced MSTBE Phases" methodology is used for the key elements of the study design used in this research, depending on data collection, approvals, techniques, and analysis methods. As a case study, these key elements are in the documentation, analysis, and development of the Muharram Bek El Mowkaf El Gedid Mobility Hub (MBMH) and the 800 m radius around it.

**Results: **The results indicate that Enhanced MSTBE Phases led to the establishment of the MBMH and the 800 m radius surrounding it as a sustainable MSTBE in Alexandria, Egypt, which is chosen as the case study.

**Conclusions: **The development of this MSTBE is a catalyst for future effects that will have a long-term impact on meso-scale and, ultimately, macro-scale transit built environments.

## Introduction

**Figure 1.**
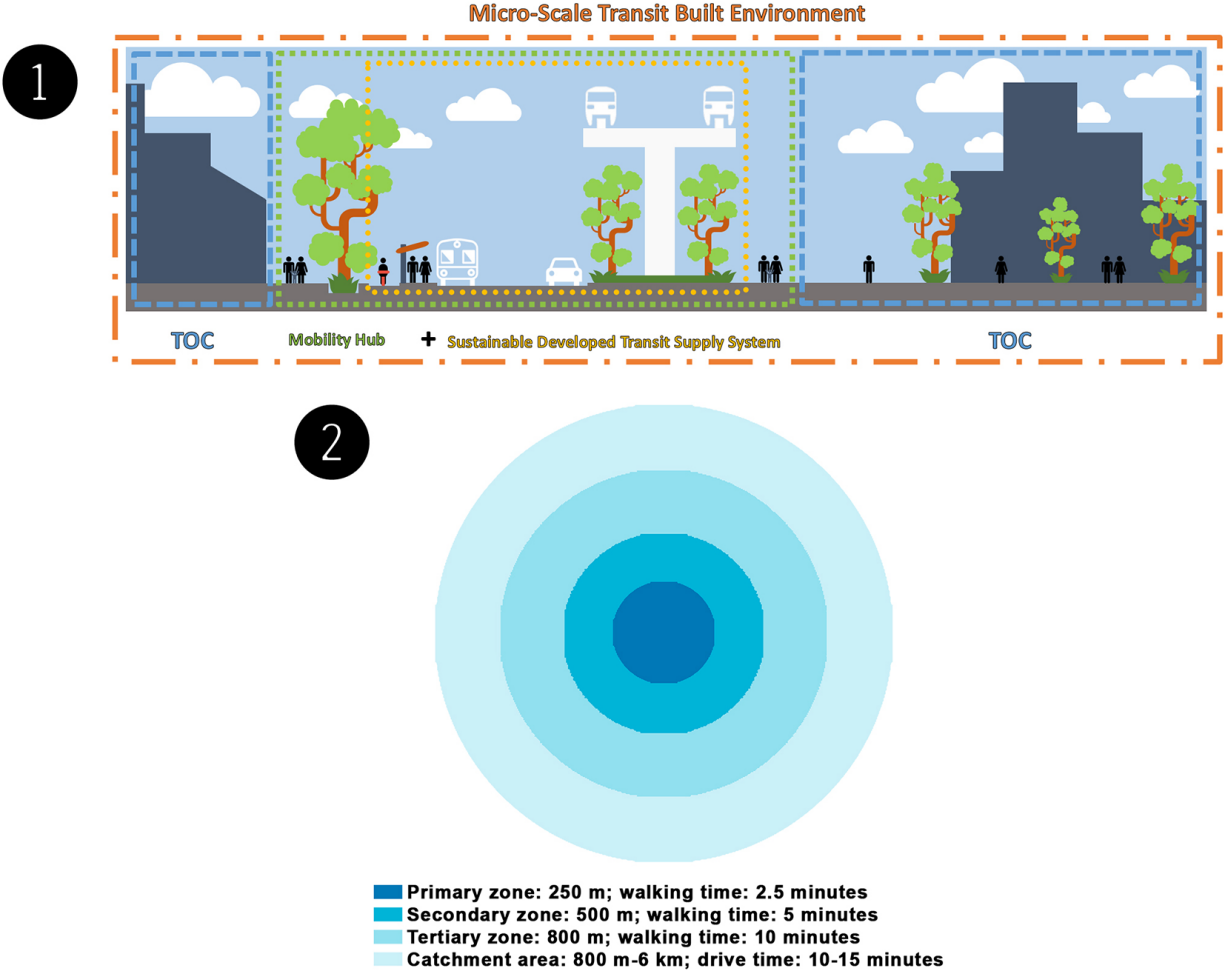
(1) A micro-scale transit built environment includes transit-oriented communities, a sustainably developed transit supply system, and a mobility hub. This figure is the authors’ own work. (2) Mobility hub zones includes the primary zone, secondary zone, tertiary zone, and catchment area. This figure is the authors’ own work.

A micro-scale transit built environment (MSTBE) refers to neighbourhood-specific urban design that integrates relevant built environment and transportation indicators. It is the smallest scale since it deals with internal trip capture, relative friction, and the pedestrian environment. ^1^ The authors split the sustainable MSTBE into three scopes: transit-oriented communities (TOCs), sustainably developed transit supply systems, and the mobility hubs (including their zones), all of which are supported by transit frameworks, strategies, solutions, and guidelines (see Figure 1-1). The authors derived these three scopes from two international case studies in Canada and the United Kingdom (see Data collection, Methods). From these international case studies, the authors have derived lessons that will be useful for the research’s case study development phase.

TOCs are walkable, compact, and well-connected neighbourhoods. These neighbourhoods are intended to encourage active transportation by focusing on high-density, mixed-use, and pedestrian-friendly development within the walking distance of frequent transit and utilising mobility management strategies to reduce unnecessary driving. TOCs must be regarded as part of the government's attempt to establish sustainable and innovative means of transportation. The TOC approach aims to aid in developing communities based on transit stations to increase ridership, alleviate traffic congestion, and enhance housing and job opportunities. All of this contributes to the development of entire communities based on solid urban planning and design guidelines. Collaborating to create TOCs has the following primary advantages: the building of complete communities, added value, the creation of a mix of uses, a vibrant public realm, and prominence. ^2^^–^^4^

Transit infrastructure and transit services are components of a sustainably developed transit supply system. Transit infrastructure consists of the basic facilities, structures, equipment, technologies, and services that support economic activity and quality of life. It promotes local and regional development by facilitating the flow of goods, connecting production centres with markets, and facilitating people's movements by providing access to work, social opportunities, health and educational facilities, and other services. At the same time, transit services encompass all services (sea, air, land, inland waterways, surveying, and pipelines) that involve the movement of people and products (freight), the rental of carriers with a crew, and related support and auxiliary services – the type and quality of transit services in a neighbourhood influence the establishment of a TOC. Transit service types can be selected based on speed reliability, regardless of infrastructure, and local access attributes are primarily determined by the right-of-way (ROW) type and station or stop spacing.

**Figure 2.**
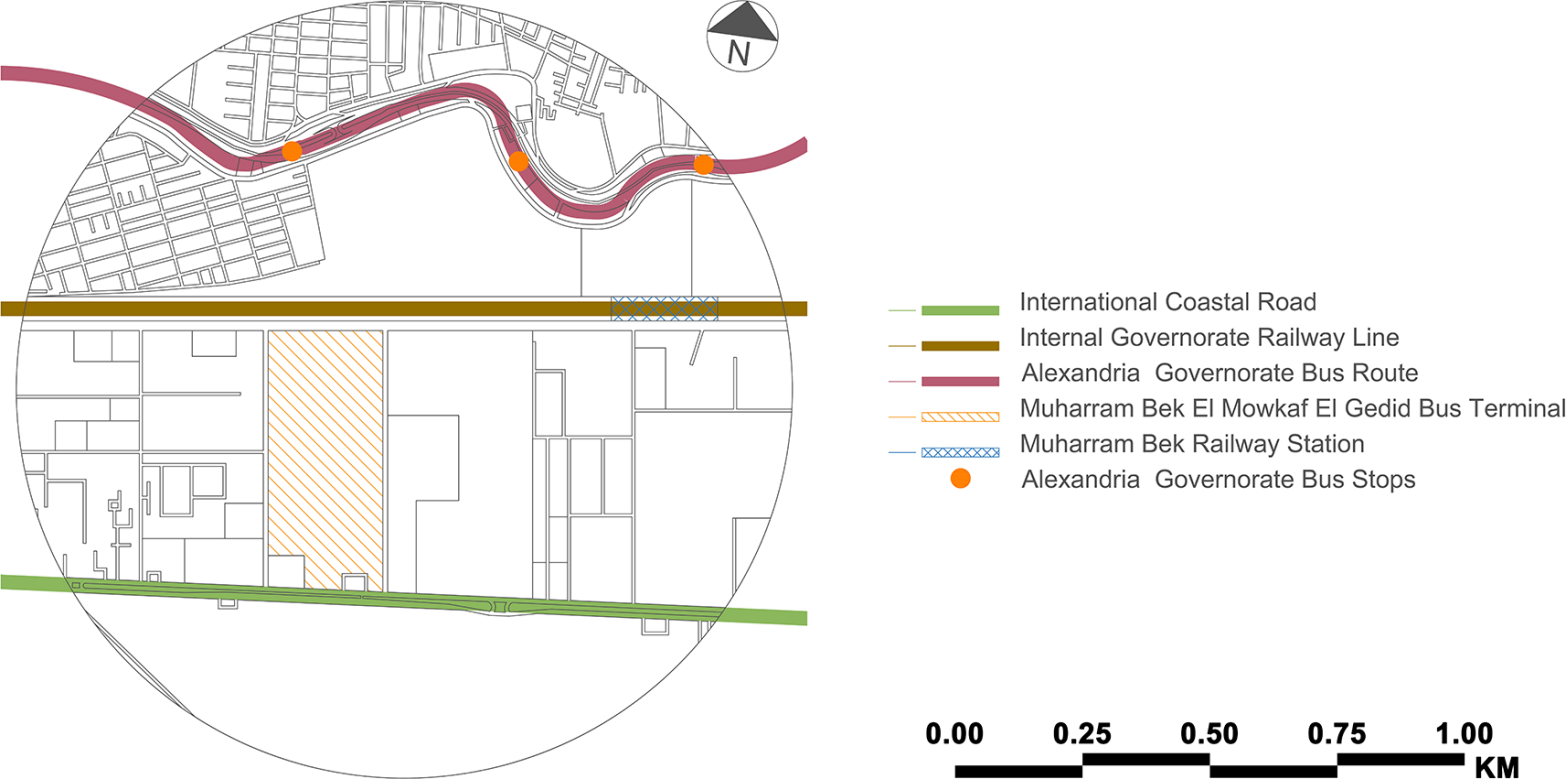
Public transit modes (lines/routes/stops/stations) – case study zone (present). This figure is the authors’ own work.

A mobility hub is a multimodal transportation centre that includes major transit stations and their surrounding areas (approximately 10 min/800 m radius), that connect transit, active transportation, and car commutes and that increase the use of shared modes with an emphasis on employment, living, shopping, and/or recreation. This type of hub is frequently seen as a location where new transportation technology and services can be integrated and utilised to improve user experience and increase transportation alternatives for first- and last-mile travel. As the origin, destination, or transfer point for many trips, a mobility hub plays a vital role in the regional transit system. Mobility hubs come in a variety of shapes and sizes, ranging from city centre hubs to suburban small stations, from those directed to rural environments to those oriented towards tourism. ^5^^–^^7^ Collaborative Mobility UK (CoMoUK) developed Mobility Hub Accreditation to provide a set of guidelines for evaluating the quality of mobility hubs. The guidelines include six criteria for effective scheme design: visibility and accessibility, sustainable mode selection, safety, ease of switching modes, practical amenities, and social and community appeal. When planning the process and understanding the needs and potential in each area, it is common practice to split a mobility hub into zones. The four zones are the primary zone, secondary zone, tertiary zone, and catchment area (see Figure 1-2). ^8^

## Research problem

There have recently been statements that Alexandria's ongoing and future transportation projects will be implemented, such as the high-speed train El Ain El Sokhna-Marsa Matrouh, the Alexandria Metro (Abu Qir-Misr Station), Raml Tram Rehabilitation, and the Establishment of Central Stations on the Express Train Track. These statements are on the official website of the Egyptian National Authority for Tunnels. ^9^^–^^12^ From the perspective of the authors, accepting these projects is unfeasible because the existing MSTBEs all over Alexandria are unsustainable owing to their lack of scopes: TOCs, sustainably developed transit supply systems, and mobility hubs. Even if there are one or two scopes, the MSTBE will be incomplete.

According to many facts, the Alexandrian MSTBE is incomplete. First, current Alexandrian TOCs are imperfect communities based on poor design principles and have no sustainable guidelines to guide the urban design of qualified transit stations and their surroundings, which results in passenger discomfort. Passengers encounter problems in direct and indirect ways, and therefore, passengers are averse to using public transportation and are forced to use private cars. Second, there are delays in receiving new transit supply system technologies that are currently being developed or that are expected to be implemented over the next five to ten years as a consequence of the authorities’ faltering. Lastly, each mode of public transportation operates independently, without integration with other transit systems, and does not facilitate passengers’ movement from the start to the end of their trips.

## Study aim

The study’s aim is to redirect authorities’ focus from strategic plans to the existing MSTBEs of cities. This research establishes a sustainable urban design for MSTBE by gathering, studying, capturing image survey data on the current state of the case (see Data availability ^13^), and using SWOT analysis to create a seamless integration built environment in an 800m radius around various transit mode intersections, provide arrival and departure passengers with mobility comfort throughout the hierarchy of access, and promote a shift in travel behaviour to the greatest extent possible through relevant decision-making, such as transit system planning, design, financing, and operation. Another aim is to create a method that includes phases supported by transit frameworks, strategies, solutions, and guidelines to establish an 800 m radius micro-scale transit built environment.

The interaction and balance between transportation, land use, and place-making functions were used throughout to meet the objectives of the study, including the following:

• Establishing TOCs, which, by design, encourage people to drive less, walk, cycle, and cross more, with a concentration on high-density mixed-use development on a human scale around frequent transit stops and stations.
• Developing an environmentally friendly transportation supply system.
• Creating a mobility hub to merge several forms of transportation into a single focal point.

## Methods

### Ethical approval and consent

Our research paper has no relevance to studies involving humans (individuals, human data, or material) or human participants, including personal genomics studies or clinical trials. Therefore, the authors are not concerned with the Helsinki Declaration. Any individual or attendant from any of the institutions visited during this research does not necessitate any protocol or consent from the individual for the use and publication of data in Egypt. Ethical approval must be obtained only when information is not open access.

### Study design

The “Enhanced MSTBE Phases” methodology was created by the authors to establish a sustainable MSTBE, including documentation, analysis, and development. This methodology was the one that was used for the key elements of the study design used in this research, depending on data collection.

**Approval requests:** Formal requests were made for information and approvals to the transport institutions to obtain approval to use and share the data and not to face any objections throughout the research operation.

**Techniques:** The techniques involved looking for documents, maps, manuscripts, and references related to transportation in Alexandria and photographing the case study site by using a digital single-lens reflex (DSLR) camera with a professional camera tripod, a padcaster tripod dolly wheel, and a smartphone with a DJI Osmo Mobile 3 Gimbal for the smartphone.

**Analysis Method:** SWOT analysis was used as a tool to determine the strengths, weaknesses, opportunities, and threats of the case study site.

### Settings

The study focused on the MSTBE in Alexandria and highlighted the Muharram Bek El Mowkaf El Gedid Mobility Hub (MBMH) and the 800 m radius surrounding it as the case study. The authors established a proposal for the MBMH and the 800 m radius surrounding it. This study used the “Enhanced MSTBE Phases” methodology, which was created by the authors, to establish a sustainable MSTBE, including documentation, analysis, and development.

### Data collection

Data collection depended completely on the documentation phase, which was the first phase where data and information were collected. From this phase, the authors were able to complete the next phases. The first phase was documentation, which included researching and gathering information and data on the current state of the case. This phase was divided into four aspects, including visits, photography, drawings, and TV shows. The second phase included conducting an overall analysis of the data collected in the first phase, utilising SWOT analysis. Depending on the two prior phases, the third phase came with a proposal for the case study.

Also, the authors used two international case studies’ lessons, as well as data collection, about what needs to be done to move towards sustainability and completion in a MSTBE: the Kennedy Station Mobility Hub in Toronto, Canada, and the London King’s Cross in London, United Kingdom.

The Kennedy Station Mobility Hub highlighted the development of strong public areas that integrated numerous means of transit. We picked out the following lessons from it:

• Long-term phasing strategies should ensure that current infrastructure does not impede future development opportunities.
• Develop a coherent design language for the station site by incorporating the landscaping strategies developed for public spaces and plazas around the station into the public realm treatment of other station amenities such as the bus terminal, stops, and passenger pick-up and drop-off areas.
• Street improvements provide vital neighbourhood connections to and from the station.
• Continuous cooperation of all stakeholders with the competent authorities has a great impact on the streets’ placement and layout.
• Connecting existing roads with new roadways is necessary to make development plots accessible and provide a more continuous, grid-like road layout in general.
• Consider some unique options in mobility hubs to mitigate these visual impacts due to the inconvenience associated with the overall development, train corridors, bus traffic, and security gates.

London King’s Cross’s Regeneration Programme involved converting a community in central London into a mixed-use urban regeneration project and showed how rail companies can increase real estate market value near a central railway hub by improving connectivity, public space, public-private partnerships (PPPs), and public consultation. We picked out the following lessons from it:

• Any development requires long-term commitment and flexibility from all parties to reflect and respond to evolving market needs.
• Active community involvement ensures stronger stakeholder engagement and better outcomes.
• Market value can be created around well-connected hubs with great market potential and quality public spaces.
• Keep the PPP concept in mind, which entails financing infrastructure and property development as well as building and operating various projects to achieve a compromise between the developer’s long-term goals of creating and managing an estate and the local government’s desire to incorporate development into the communities that surround it.

### Documentation phase

As mentioned earlier, this phase was divided into four aspects:

**Visiting relevant transport institutions:** Visits were made to the General Authority for Passenger Transport in Alexandria, Public Authority for Planning Transport Projects, National Railway Authority of Egypt, Ministry of Transportation, and Directorate of Housing and Utilities–Alexandria. Appointments were made:

• To view documents, maps, manuscripts, and references related to transportation in Alexandria, such as the Strategic General Urban Plans, which were prepared by the Egyptian General Organisation of Physical Planning (GOPP) in collaboration with other authorised accredited local and foreign organisations: the Maclean Plan 1921 and General Plan (GP) 1959, GP 2005, GP 2017, GP 2025, GP 2032, and GP 2050 (the authors have collected the plans into two figures; see Data availability, ^13^ “Strategic General Urban Plans (1).jpg” and “Strategic General Urban Plans (2).jpg”).
• To have some available public notes for the transit projects in Alexandria that may be helpful in the study, such as titles of ongoing projects and whether they are on schedule i.e., the high-speed train El Ain El Sokhna-Marsa Matrouh, the Alexandria Metro (Abu Qir-Misr Station), Raml Tram Rehabilitation, and the Establishment of Central Stations on the Express Train Track, as the Egyptian National Authority for Tunnels announced about them; the Alexandria urban transport study is financed by the neighbourhood investment facility from the European Union and managed by the French Development Agency (Agence Française De Développement); the Egyptian Government commissioned EGIS rail, a French advisory office, to prepare a long-term scenario for the Alexandria governorate strategic plan for urban transportation consistent with urban planning for the city; and a partnership between SYSTRA, AECOM, Orascom Construction SAE, The Arab Contractors, Siemens Mobility, and other companies with the Egyptian ministries to consult, design, install, commission, and maintain the systems for the projects.
• To facilitate the operation of capturing image survey data on the current state needed for the case study.

Prior to the visits, the authors submitted “Formal Requests for Information and Approval”, signed by the Vice Dean for Graduate Studies and Research, Prof. Sahar Mahmoud Al-Arnaouti, and stamped by the Faculty of Fine Arts, to the transport institutions and obtained approval to use and share the data.

For the copies and translation of the formal requests for information and approval, see Data availability. ^13^

**Capturing image survey data:** The authors used the following photography and panoramic photography tools to acquire image survey data: (1) a DSLR camera with a professional camera tripod, (2) a padcaster tripod dolly wheel, and (3) a smartphone with a DJI Osmo Mobile 3 Gimbal for the smartphone to capture image survey data on the current state of the case study (see Data availability, ^13^ “00 Capturing Image Survey Data Tools.jpg”, “01 Primary Zone.jpg”, “02 Secondary Zone.jpg”, and “03 Tertiary Zone.jpg”).

**Overlaying, tracing, drawing, and presenting:** The authors used the maps obtained from the institutional archives (the authors have collected the plans into two figures; see Data availability, ^13^ “Strategic General Urban Plans (1).jpg” and “Strategic General Urban Plans (2).jpg”) and the exported images from Google Earth Pro (see the software availability statement for alternatives) to draw the new Alexandria Transit Map by overlaying and tracing with Autodesk AutoCAD (see the software availability statement for alternatives). Then, the authors cropped the case study zone from it and presented the case study zone using Adobe Photoshop (see the software availability statement for alternatives).

**Transcribing announcement on TV shows:** On the “Al Hekaya” TV talk show, presenter Amr Adeeb and Kamel El-Wazir, the minister of transport of Egypt, announced official transportation news about the transit developments in Egypt and Alexandria (see Data availability, ^13^ “Official Transportation News through the Media.pdf” provides descriptions of the news).

After conducting comprehensive research and data collection, the authors selected the MBMH as the case study zone from among 23 mobility hubs indicated on the new Alexandria Transit Map. The case study zone is located in Alexandria's core area and includes the 800 m radius surrounding the Muharram Bek El Mowkaf El Gedid Bus Terminal. Before the 1952 revolution, the Muharram Bek neighbourhood was considered an elite neighbourhood, with mansions and villas dominating the area. In accordance with GP 2032, an electric high-speed rail terminal will be constructed in front of the Muharram Bek El Mowkaf El Gedid Bus Terminal. Table 1 presents the current profile for the case study zone.

**Table 1.** Current profile – case study zone (present).

Item	Description
**Location**	The 800 m radius surrounding the Muharram Bek El Mowkaf El Gedid Bus Terminal
**Infrastructure – Lines and Routes**	• International Coastal Road • Alexandria Governorate Bus Route • Internal Governorate Railway Line
**Infrastructure – Others**	• Martyr Soldier/Muhammad Reda Muhammad Ahmad Bridge • Stations/stops: Muharram Bek El Mowkaf El Gedid Bus Terminal and Muharram Bek Train Station • Control systems • Support • Guidance • Propulsion • Control
**Transit Modes**	• Regional and national buses • Alexandria Governorate buses • Private cars • Taxis
**Non-Mobility and Urban Realm Improvement Mobility Components**	Covered waiting area

The Muharram Bek El Mowkaf El Gedid Bus Terminal is the new bus terminal for regional and national bus connections located in Alexandria's Muharram Bek area. Construction of it began in 2001 and was completed in 2003. It is operated by the National Company for Road Construction and Development and serves as the primary departure and arrival point for passengers travelling by land to and from Alexandria. It was considered an alternative to the old bus stop at the Sidi Gaber Train Station before all buses and taxis for the governorates were relocated to the present location. Public transportation runs across the case study zone (see Figure 2). However, there is no integration between two or more public transportation modes. Arriving passengers could use the train, but since the current Muharram Bek railway station is more than 1 kilometre distant, they cannot. An extra stop along the new suburban train line would be beneficial, and getting to the Misr Station Train Station is also difficult. Although the Misr Station is approximately 2 kilometres distant, the railway line prevents pedestrian access to the city centre.

### Analysis phase

**Figure 3.**
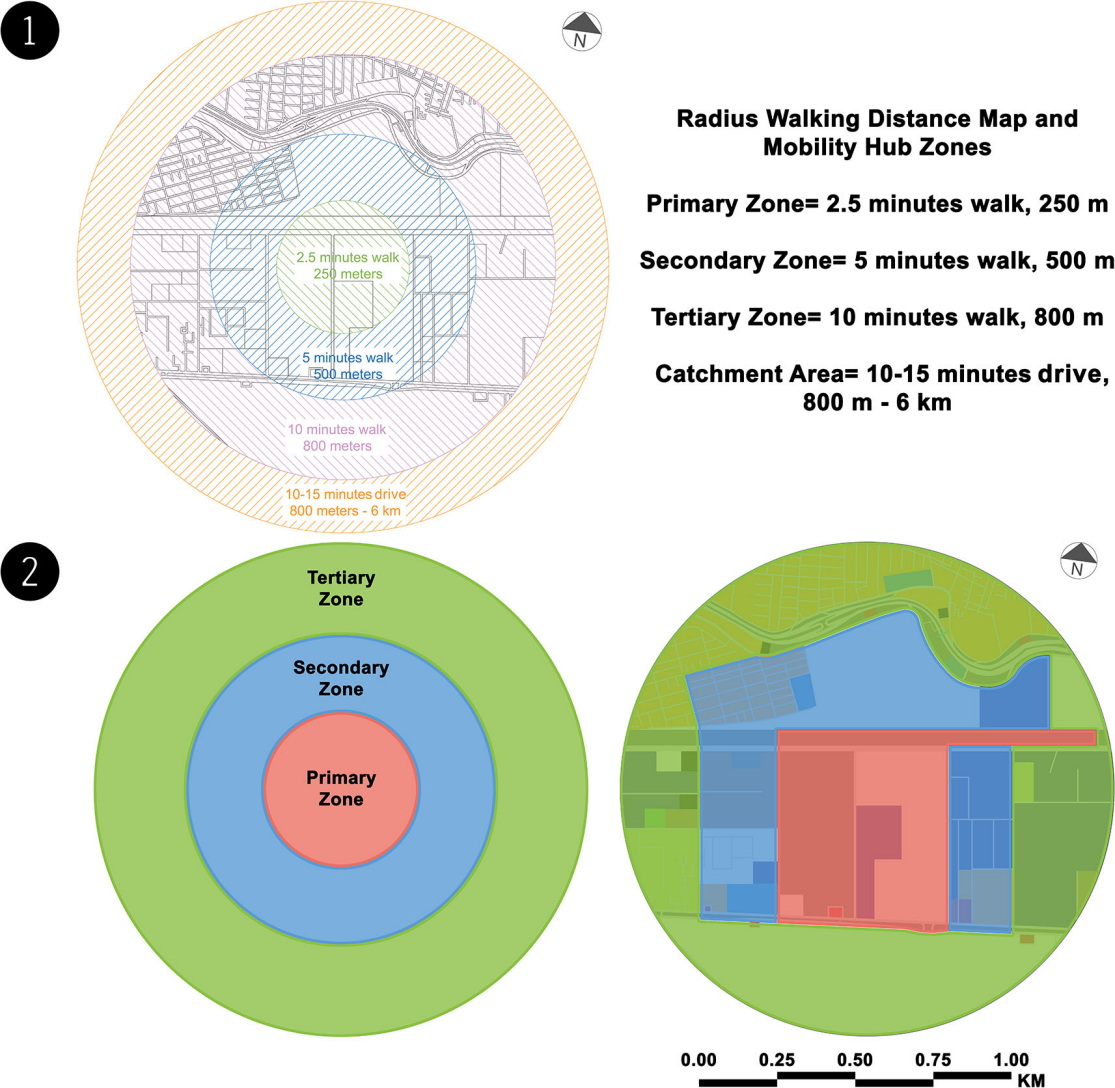
(1) Radius walking distance map – case study zone (present). This figure is the authors’ own work. (2) Mobility hub zones – case study zone (present). This figure is the authors’ own work.

There is currently no mobility hub in the case study. However, the authors created a radius walking distance map to identify and analyse the mobility hub zones (see Figure 3-1). The following table (see Table 2) shows the SWOT analysis for each mobility hub zone (see Figure 3-2), with reference to Table 1. SWOT analysis is used to investigate the current and initial conditions of the planned hub site and its surroundings. Analyse existing transport networks, including street connections, cycling, pedestrian infrastructure, and public transport. This review included land use, urban form, and neighbourhood character to fully capture the context of the site. Redevelopment opportunities on and around the site were analysed to understand the potential of mobility hubs to support transit-oriented development. Finally, the site's constraints and opportunities are discussed to determine which elements of the mobility hub are best suited to the site's current issues and build on its strengths. In addition, the view of documents, maps, manuscripts, and references related to transportation in Alexandria, notes taken from visiting relevant transport institutions, and El-Wazir's explanation of official statements from transcribing announcements on TV shows helped the authors build the outline description of the current situation for the SWOT analysis.

**Table 2.** SWOT analysis of mobility hub zones.

SWOT	Primary Zone	Secondary Zone	Tertiary Zone
**Strengths**	• There are lighting units along the road • Traffic lights control the vehicle and pedestrian passage beside the bus terminal in both directions • There are street signs • There are taxicabs • The Muharram Bek El Mowkaf El Gedid Bus Terminal has an administration building, covered seating areas, bathrooms, and a ticket office • The bus terminal and its platforms are generally in good condition and clean • There are paid parking spaces and commercial shops inside the bus terminal • The Muharram Bek Train Station contains a covered seating area and an administrative building with a manager's office, a bathroom, a computer desk, and a ticket office • The train station and its platforms are generally in good condition and clean	• There is a parking district – West Alexandria • There is a car dealership (show and sell) • There are government buildings and institutions	• There is a pedestrian connection, i.e., the Martyr Soldier/Muhammad Reda Muhammad Ahmad Bridge • Residential buildings with heights ranging from 4 to 12 floors • There are development areas; each of them includes a mosque, an event centre, playgrounds, a wedding hall, a swimming pool, and a commercial centre • There is sporting club • There are government buildings and institutions
**Weaknesses**	• Entering the Muharram Bek Train Station by an improper entry; on the other hand, the main gate is sealed • The Muharram Bek El Mowkaf El Gedid Bus Terminal is not visible coming from the El Kabary Express Bridge • Private vehicles and taxicabs are parking against the bus terminal's wall, causing traffic congestion • There are no stable taxi stands, which causes traffic problems • The minibuses are causing traffic congestion at traffic lights by stopping illegally • No bus lanes • No safe access to bus stops, which must be provided through sidewalks and proper street crossing sites • At bus stops, there are no agency logos or visual markers, station names, route maps, or schedules, all of which should be displayed to riders • The presence of three-wheel tuk-tuks causes traffic problems • There is no pedestrian infrastructure that ensures pedestrian safety • There are no crosswalks where the roads would be too unsafe to cross without assistance due to vehicle numbers, vehicle speed, or road widths • No cycling facilities • No cycle tracks • Cyclists cannot ride safely in mixed traffic • No shared modes, either car or bike • No alternate parking spaces with trees or rain gardens • There are no open spaces • There is no balance in the distribution of services depending on the building types on the site • There are street vendors in the surroundings • The lighting units along the road do not usually work at night	• No vehicular infrastructure serves the Muharram Bek Train Station on both sides of the train station • There is no pedestrian infrastructure that ensures pedestrian safety • There are no crosswalks where the roads would be too unsafe to cross without assistance due to vehicle numbers, vehicle speed, or road widths • No cycling facilities • No cycle tracks • Cyclists cannot ride safely in mixed traffic • No shared modes, either car or bike • No alternate parking spaces with trees or rain gardens • There are no open spaces • There is no balance in the distribution of services depending on the building types on the site	• Building heights are inconsistent with street widths, and there are various heights on both sides of the Mahmoudia Canal Road • There is no pedestrian infrastructure that ensures pedestrian safety • There are no crosswalks where the roads would be too unsafe to cross without assistance due to vehicle numbers, speed, or road widths • At intersections, there are no raised crosswalks, which act as speed calming measures and prioritise pedestrians • No alternate curb extensions or rain gardens with parking spaces to create pinch points on the streets, which help in speed reduction • No curb extensions to locate street trees, light poles, cycle racks, or other street furniture • No cycling facilities • No cycle tracks • Cyclists cannot ride safely in mixed traffic • No shared modes, either car or bike • No alternate parking spaces with trees or rain gardens • There are no open spaces • There is no balance in the distribution of services depending on the building types on the site
**Opportunities**	• The Alexandria High-Speed Electric Rail Terminal will be built on the vacant site in front of the Muharram Bek El Mowkaf El Gedid Bus Terminal; a sign confirms the existence of the Egypt Electric High-Speed Rail Project. The Egyptian Ministry of Transport owns the project, which is supervised by the General Authority for Roads, Bridges, and Land Transport; the general consultant for the project will be SYSTRA, and CASA Construction will handle the contracting; this project will cause a qualitative shift in the realm of transportation, affecting the MSTBE • There are development services in front of the bus terminal, i.e., petrol and gas stations with commercial amenities located in front of the bus terminal	• The vacant site on which the Alexandria High-Speed Electric Rail Terminal will be established is far too large to be managed alone; the authors expect that further projects will be planned on this land to serve and support the terminal • There are development services, i.e., petrol and gas stations and a car service centre	There are development services, i.e., petrol stations, car service centres and a supermarket
**Threats**	There is no provision to guarantee the safety of people crossing the train track from mixed-use land		Pedestrians do not generally use bridges except a few times due to the many stairs on the bridges; pedestrians jump over concrete barriers to pass from one side to the other, often exposing passers-by to danger; some neighbourhood residents made a small ladder bypassing by jumping or using bridges

### Development phase

The authors built a vision of development for MBMH to become a mixed-use attraction within the city with a new profile (see Table 4). In Table 3, the authors worked on developing certain multi-dimensional aspects, such as land use, the built form, and open space and circulation, so that they could offer recommendations for the case study zone.

**Table 3.** Recommendations for the case study zone (proposal).

Land Use	Built Form	Open Space & Circulation
• Concentrate mixed-use development along major transit corridors • Emphasise mixed-use infill on unoccupied and unused lots • When employment objectives are met, explore additional applications to create a vibrant hub • Investigate partnership opportunities for large-format commercial usage • Concentrate the highest density near transit services • Provide proper transitions to nearby stable residential neighbourhoods • Provide necessary services while keeping residential building heights and open areas in mind	• New developments should support improved transit services • Maintain visible and physical connections to the station while new development occurs • Provide mid-rise buildings to concentrate the maximum height and density near the rail corridor • Integrate station amenities on both sides of the transit corridor to provide a walkable environment with direct, weather-protected access	• Consolidate access and servicing to new development • Implement living streets on the roadways surrounding the transit corridors • Design buildings on large development blocks to frame outdoor areas (parks, courtyards, gardens, parklets) to give views of the station and to enable continuous access between sites • Create new open spaces within significant developments • Create new cycling facilities • Encourage pedestrian-friendly street design on the roadways near the transit corridors • Make safe and direct connections across the transit corridors

**Table 4.** Muharram Bek El Mowkaf El Gedid Mobility Hub – future mobility hub profile – case study zone (proposal).

Item	Description
**Mobility Hub's Name**	Muharram Bek El Mowkaf El Gedid
Context	Suburban mixed: • ADA accessible design • Pedestrian access • Bicycle access • Parking/ park & rides • Passenger loading zones • Car-share access • Bike-share access • Micro-mobility access • Real-time transit information • Integrated trip planning • Integrated fare payment • Wayfinding • Shelters • Benches • Lighting • Services & retail • Hub placement
**Transport Function**	Destination
**Type**	Anchor
**Scale**	City centre mobility hub
**Infrastructure – Lines & Routes**	• International Coastal Road • Alexandria Governorate Bus Route • Internal Governorate Railway Line • Mahmoudia BRT Route • Mina El Basal – Muharram Bek BRT Route • Egypt Electric High-Speed Rail Line
**Infrastructure – Others**	• Walkways/pedestrian connections • Bridges supported by escalators and elevators • Stations/stops: Muharram Bek Railway Station (after relocation), Bowalino BRT Stop, Alexandria High-Speed Rail Station BRT Stop, Muharram Bek El Mowkaf El Gedid BRT Stop, Alexandria High-Speed Rail Terminal, Muharram Bek El Mowkaf El Gedid Bus Terminal and Alexandria Governorate Bus Stops • Control systems • Support • Guidance • Propulsion • Control • Intelligent transit systems • Transportation demand management • Cycling infrastructure • Living streets
**Transit Modes**	• Regional and national buses • Alexandria Governorate buses • Private cars • Taxis • Internal Governorate Railway • Mahmoudia BRT • Mina El Basal – Muharram Bek BRT • Egypt Electric High-Speed Rail • Pedestrian connections • Car club bay – electric and conventional • Bike-share – electric and conventional
**Related Mobility Components**	• Large-scale cycle parking • Digital pillar (transport info, ticketing, wayfinding, walking distances, local services) • EV charging bays
**Non-Mobility and Urban Realm Improvement Mobility Components**	• Covered waiting area • Improved public realm, safer crossing, road/pavement repairs • Parklet/community art • Kiosk for refreshments
**Park & Ride**	The authors propose relocating the Muharram Bek Train Station to the mobility hub site, accompanying the park and ride

In regard to the authors’ recommendations, lessons, and guidelines, the authors put the major points for the development of the drastic changes in the current state of the whole case study area based on the demands of each mobility hub zone as follows:

• In the primary zone, the authors focused on utilising this zone on the MBMH and its amenities on both sides of the transit corridors to create a distinctive mobility hub location with appealing streetscapes, reinforce the built form, and transition open space to new residential uses to offer a walkable environment with direct and weather-protected access.
• Secondary zone utilisation is mostly mixed-use development along major transit corridors with enhanced transit services and visible and physical links to the mobility hub.
• Mid-rise buildings are primarily clustered around transit services in the tertiary zone, with the greatest height and density along the railroad track. They supported by a mixed-use development that optimised connections and views.
• The needed services are provided within and around the buildings, considering the heights of residential buildings and open spaces.
• Took into account the distance between stops and stations and the smoothness of the traffic flow.
• Living streets are settled with two alternative street widths for the neighbourhood, one of which is 14 metres and the other is 20 metres.
• Cycle infrastructure is settled along transit corridors and within residential buildings, creating safe and direct connections across the transit corridors.
• Urban street elements are settled to contribute to the safety and attraction of urban streets for people walking, shopping, parking, and driving. The proposal used dedicated median bus lanes, gateways, bus stops, bike corrals, bike share stations, parklets, and crosswalks.

## Results and discussion

**Figure 4.**
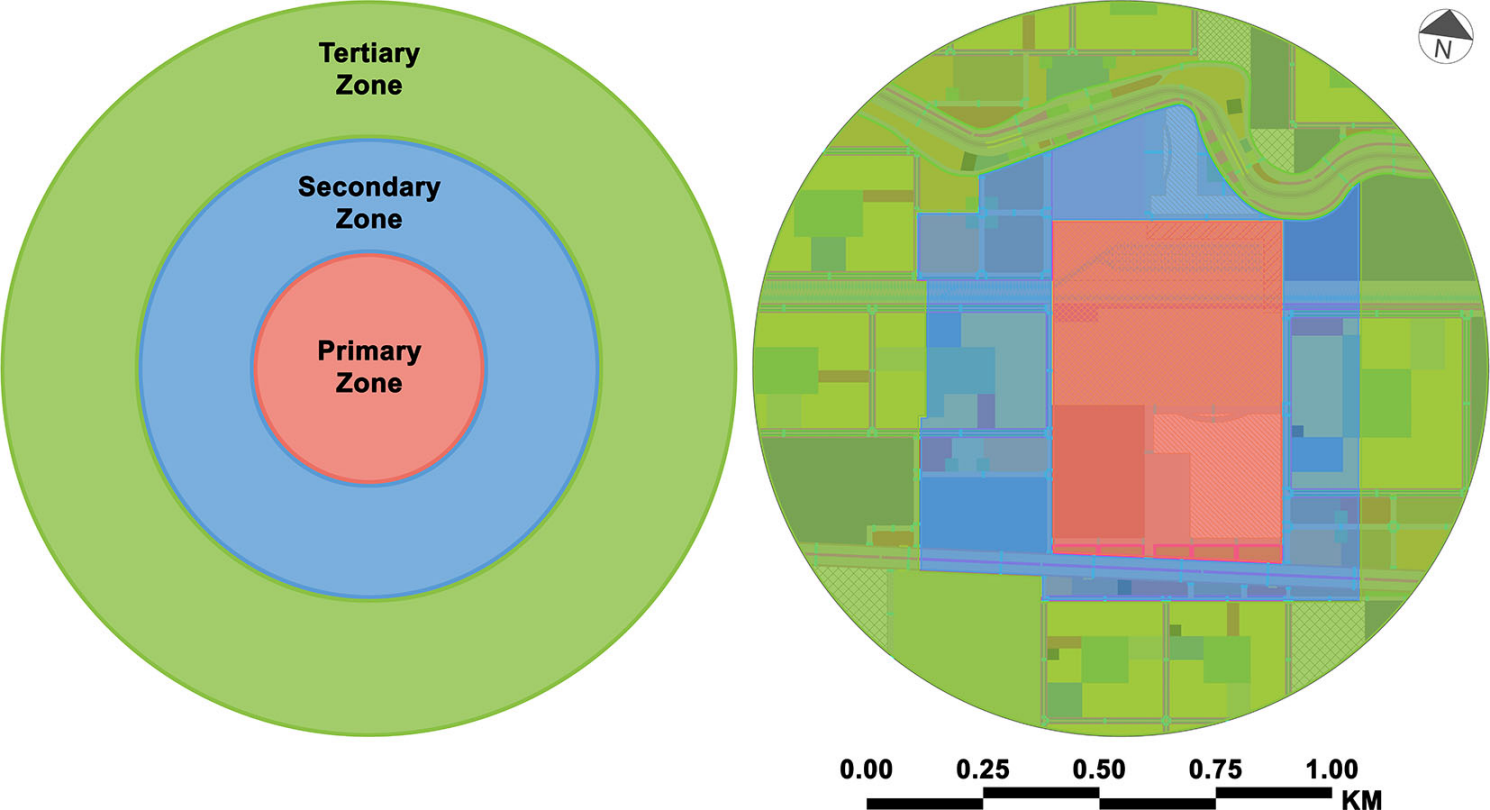
Mobility hub zones – case study zone (proposal). This figure is the authors’ own work.

**Figure 5.**
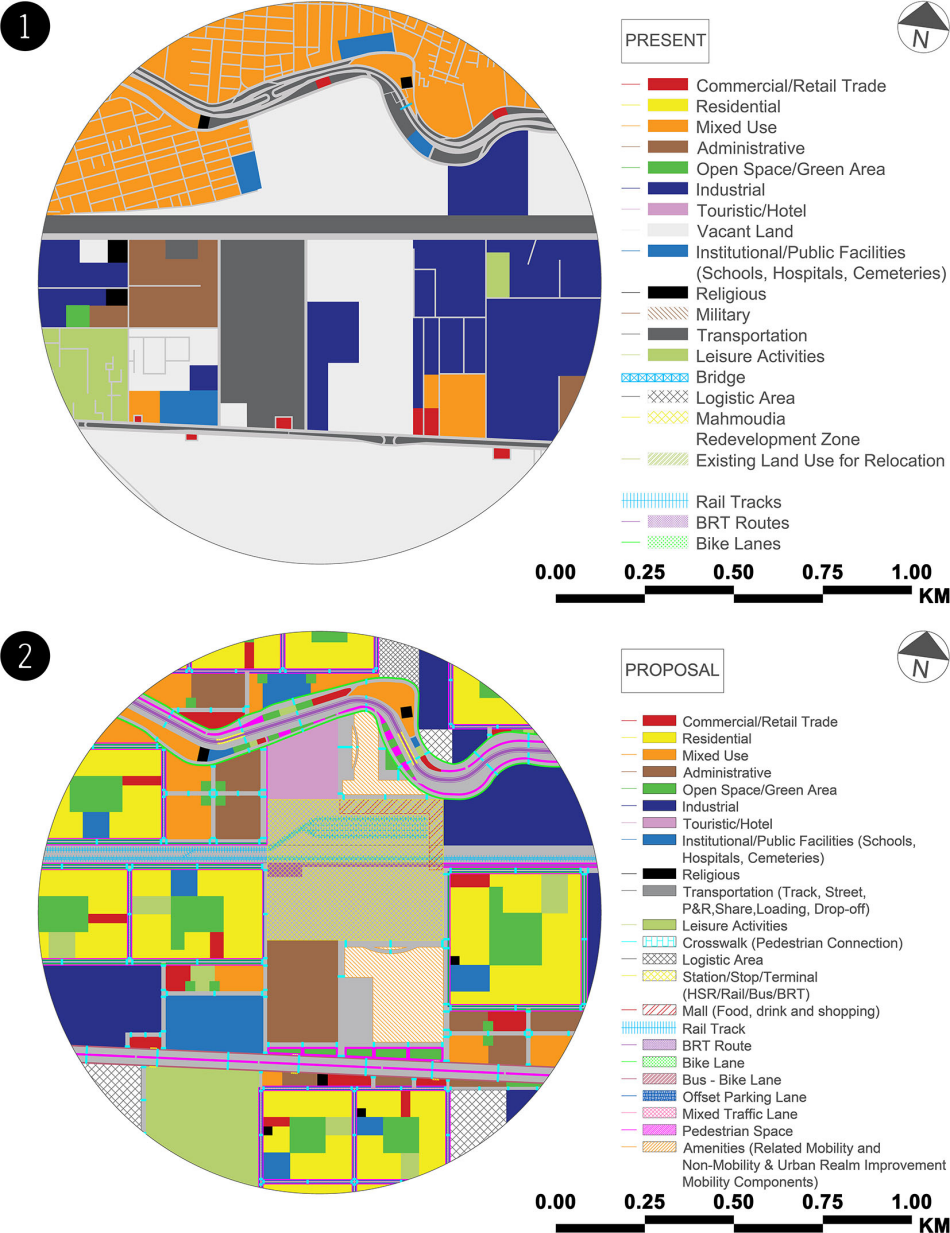
(1) Land uses map – case study zone (present). This figure is the authors’ own work. (2) Land uses map – case study zone (proposal). This figure is the authors’ own work.

As shown in Figure 4 and Figure 5-2, the authors took several phases into account, including the accumulation of lessons, information, and data and the analysis and development of the current state, in order to have acceptable results in the long run, even though there were negative results. The proposal achieved the following:

• In the primary zone:
  ○ Locating Muharram Bek Railway Station (after relocation), Alexandria High-Speed Rail Terminal, Muharram Bek El Mowkaf El Gedid Bus Terminal and Alexandria Governorate Bus Stops in this zone will be the busiest in terms of mobility.
  ○ This zone will prioritise high levels of pedestrian and transit traffic while balancing many station access routes.
  ○ This zone will encourage high levels of flow as well as maximum intensity and diversity in order to create a diverse and active hub of activity for the local population.
  ○ This zone will provide travellers with facilities through development.
• In the secondary zone:
  ○ Direct, safe pedestrian and cycling lanes will link rapid transit stations.
  ○ The high availability of public transport will facilitate sustainable mode sharing, enabling high density and diversified use.
• In the tertiary zone:
  ○ Walking, cycling, and local transportation feeder services will all grow in importance in the future.
  ○ The density and height of development will be increasingly moving towards the edge of the mobility hub.
• At the three scopes of the MSTBE:
  ○ Implementation of some of Alexandria’s ongoing and future public transportation projects will develop transit infrastructure and services, which will be supported by advanced sustainable programmes, technologies, frameworks, and guidelines to ensure their environmental, social, and economic impacts in Alexandria.
  ○ The MSTBE’s design will support frequent use within 400 metres of regular transit and 800 metres of ongoing or planned rapid transit stations and will support the adequate crossing of high- and low-density areas.
  ○ Utilisation of high-market-value creation, public-private partnerships, and long-term perspective approaches will enhance the MSTBE.
  ○ Creation of mobility hub will connect transit, active transportation, and vehicle commutes to enhance the use of shared modes. They will be frequently integrated with public, commercial, or residential amenities to create a dense urban environment.
  ○ Integrating transportation and neighbourhood development into TOCs will make commuting more accessible and faster. This approach will allow for the creation of vibrant, mixed-use neighbourhoods that are linked to mobility hubs.
  ○ The MSTBE will benefit from the use of transit passenger facility design principles and station design guidelines.
  ○ The MSTBE will include a complete network of sidewalks and other facilities that will be beneficial to all users, even people with disabilities.
  ○ Land use patterns for green areas or large-scale redevelopment sites will be created in tandem with walking, cycling, transportation, infrastructure, and services to connect communities to key commuter locations.
  ○ Offset Parking Lanes will handle high volumes of traffic and improve both stability and travel time on streets near or beyond vehicular traffic.

In addition to the proposal’s achievements, the authors wanted to ensure that they had distributed the land uses fairly, so they estimated the exact percentages of the land uses for each mobility hub zone in the case study in the current and proposed states and compared them (see Figure 5 and Table 5); the result was acceptable under the guidance of the two international case studies. And also, they evaluated the MBMH using the city centre mobility hub’s CoMoUK accreditation assessment, and the mobility hub was awarded CoMoUK Gold Accreditation (see Table 6).

**Table 5.** Land use percentages – case study zone (present and proposal).

Mobility Hub Zones	Primary Zone (%)		Secondary Zone (%)		Tertiary Zone (%)	
Land Use	Present	Proposal	Present	Proposal	Present	Proposal
**Commercial/Retail Trade**	12.05	28.33	16.55	29.49	71.41	42.18
**Residential**	0.00	0.00	0.00	19.28	0.00	80.72
**Mixed Use**	0.00	33.27	10.13	31.49	89.87	35.24
**Administrative**	0.00	44.76	65.46	33.89	34.54	21.35
**Open Space/Green Area**	0.00	7.40	0.00	26.12	100.00	66.48
**Industrial**	7.82	0.00	28.62	14.12	63.56	85.88
**Touristic/Hotel**	0.00	0.00	0.00	100.00	0.00	0.00
**Institutional/Public Facilities**	0.00	0.00	44.87	68.19	55.13	31.81
**Religious**	0.00	0.00	0.00	33.41	100.00	66.59
**Transportation**	23.89	34.47	26.11	24.55	50.00	40.98
**Leisure Activities**	0.00	0.00	0.00	3.53	100.00	96.47
**Logistic Area**	0.00	0.00	0.00	0.00	0.00	100.00

**Table 6.** Muharram Bek El Mowkaf El Gedid Mobility Hub - City centre mobility hub’s CoMoUK accreditation assessment.

City centre mobility hub												
Factor	Choice of sustainable modes (including retrofit of existing sites)	✓	Visibility & accessibility	✓	Ease of switching modes	✓	Safety	✓	Practical facilities	✓	Visual, social, community appeal	✓
**Essential Elements**	2+ high-quality public transport options	✓	Clear signage with network branded	✓	Co-located or within a waymarked 2 min walk	✓	Street lighting	✓	Indoor heated shelter	✓	Modern, clean infrastructure	✓
	One high-quality shared mobility option	✓	Information on what the hub is at the site and in the community	✓	Real-time transport information	✓	Staffing core hours	✓	Toilets	✓	Community consultation	✓
	Large scale cycle parking	✓	Located in a prominent, well-lit location	✓	Easily accessible transport timetable	✓	No hidden areas around the hub	✓				
			Safer crossing and pavement repairs	✓	Simple ticket purchase options	✓						
			Disabled access, no blockages	✓	Staff support	✓						
			Safe cycle routes	✓								
**Desirable Elements**	Space to increase range and scale of services	✓	Consideration for hidden disabilities	✓	Digital pillar (transport info, ticketing, wayfinding, walk distances, local services)	✓	Staffing 24/7	✓	Wi-Fi/phone charging	✓	Visual: e.g. greenery/parklet/statue/art	✓
	Public transport, e.g., rail, tram, bus	✓			Immediate co-location		CCTV	✓	Kiosk for refreshments	✓	Environmental: Measures to reduce impacts and increase biodiversity	✓
	On-demand buses, taxis, private hire				MaaS digital integration	✓			Water fountain	✓	Social: e.g., Notice board, play area, exercise equipment	✓
	Shared, e.g., car club bay - electric and conventional bike-share -electric and conventional	✓			Local tourism information	✓			Package delivery lockers	✓	Community: café with seating or retail, edible garden, community art, shared books	✓
	Infrastructure, e.g., large scale cycle parking, EV charging bays	✓							Freight consolidation		Potential for human interaction	✓
	Plans for/a network of hubs	✓									Shared workspace	✓
**Result**	**CoMoUK Gold Accreditation**											

Out of all the above outcomes, the overall results are positive, as the authors expect that if the proposal is implemented as intended, it will be the starting point to ensure that the micro-scale transit built environment is a catalyst for future influences that will affect meso-scale and ultimately macro-scale transit built environments in the long run.

## Conclusions

Transportation has changed dramatically in the last decade. Demographic changes, increased urbanisation, and changes in employment types and arrangements have put additional demand on existing transportation and transit networks. That is why the authors focused on the existing MSTBEs of cities to prepare Alexandria to accept ongoing and future transportation developments that will complement local transit.

This research used the MBMH as a case study as a starting point for applying “Enhanced MSTBE Phases” methodology in Alexandria, Egypt, where the case study was documented, analysed, and developed to be sustainable. Throughout the methodology’s application, the authors ensured that the development must start from the smallest scale (micro-scale) of the built environment to the next scales (meso-scale and macro-scale), as well as that people’s behaviour is always affected by the design of the built environment (MSTBE). Moreover, when the authors compared land use percentages and used mobility hub’s CoMoUK accreditation assessment, the results were positive, which indicated the authors’ success in the methodology’s application.

Following the successful methodology’s application to the case study, the authors anticipate that if this method is applied to the other 22 proposed mobility hubs at MSTBEs, the influences of the MSTBEs will reach the meso- and macro-scales. Moreover, Alexandria Strategic General Urban Plans’ Transportation Sector Five Goals (Support Decentralised Concentration, Improve Quality of Life & Environmental Quality, Stimulate Successful Socio-economic Development, Promote Cultural and Architectural Heritage, and Support Planning Policies for Implementation) would be achieved, which would affect the whole governorate’s urban planning and housing, urban and economic development, transportation, and the environment in the future, as shown in Table 7.

**Table 7.** The expected impacts resulting from achieving the Alexandria Strategic General Urban Plans’ Transportation Sector Five Goals.

Item	Future expected impacts
**Urban Planning and Housing**	• The governorate will be able to connect its eastern and western regions through the expansion of catchment areas, provide effective public transportation to the majority of its citizens, improve mobility in general, and improve the quality of life in the entire governorate, including Borg El Arab and future expansions. • Enhancing transit connections will ease travel to secondary regional cities.
**Urban and Economic Development**	• Designing networks will be coherent with the existing centres and urban planning. • Economic centres will be developed around mobility hubs or public transportation stations. • There will be motivation to build up international projects as attraction points along the primary routes and lines in the governorate.
**Transportation**	• Forming partnerships between private and public national and international organisations will aid in the rapid expansion and strengthening of environmentally friendly public transportation systems. • Prioritizing public transport in terms of space, time, and finances will show that public transport is faster and more convenient than driving private cars. • Promoting non-polluting modes will help reduce air and noise pollution. • The Alexandria Governorate can be marketed as a tourist destination by improving public transportation in tourist areas, connecting the airport to the city, improving the quality of life in the historic city, and coordinating transportation infrastructure with historic sites.
**Environment**	Creating open and green spaces to provide recreational areas for residents will help to enhance the beauty and environmental quality of neighbourhoods.

## Software availability

• Google Earth Pro free alternative: OpenStreetMap.
• Autodesk AutoCAD free alternatives: FreeCAD and LibreCAD (2D).
• Adobe Photoshop free alternatives: GIMP and Krita.

## Data Availability

Mendeley: Comprehensive Database for Mobility Hub Zones, 800 m radius surrounding MBBT, Alexandria, Egypt. https://data.mendeley.com/datasets/k8hybkvsb3/5. ^13^ This project contains the following underlying data:
• 01 Primary Zone.jpg (A figure shows image survey data in the primary zone = 2.5 minutes walk, 250 m. The map and the photos are acquired from the authors’ work). • 02 Secondary Zone.jpg (A figure shows image survey data in the secondary zone = 5 minutes walk, 500 m. The map and the photos are acquired from the authors’ work). • 03 Tertiary Zone.jpg (A figure shows image survey data in the tertiary zone = 10 minutes walk, 800 m. The map and the photos are acquired from the authors’ work). • Official Transportation News through the Media.pdf (A PDF file provides descriptions for official transportation news through the media). • Strategic General Urban Plans (1).jpg:
‐ Map 1921: This figure has been reproduced from public domain available content from New York Public Library [URL link: https://digitalcollections.nypl.org/collections/city-of-alexandria-town-planning-scheme#/?tab=about]. ‐ Map 1958: This figure has been reproduced from public domain content available from Wikimedia Commons [Licence: US Army Corps of Engineers, 1959 map of Alexandria, Egyptian Region United Arab Republic, CC0 1.0, URL link: https://commons.wikimedia.org/wiki/File:1959_map_of_Alexandria,_Egyptian_Region_United_Arab_Republic.jpg]. ‐ Map 2005: This figure has been reproduced and publicly shared with the permission from City’s Municipal of Alexandria. ‐ Map 2017: This figure has been reproduced and publicly shared with the permission from General Authority for Urban Planning. ‐ Map 2025: This figure has been reproduced and publicly shared with the permission from Advisory organisation for implementation of comprehensive planning for Alexandria. ‐ Map 2032: This figure has been reproduced and publicly shared with the permission from General Authority for Urban Planning.
• Strategic General Urban Plans (2).jpg (The authors obtained permission to publicly share this figure from City’s Municipal of Alexandria). Mendeley: Comprehensive Database for Mobility Hub Zones, 800m radius surrounding MBBT, Alexandria, Egypt. https://data.mendeley.com/datasets/k8hybkvsb3/5. ^13^ This project contains the following extended data:
• 00 Capturing Image Survey Data Tools.jpg (A figure shows the capturing image survey data tools used in the survey - (1) a DSLR camera with a professional camera tripod, (2) a padcaster tripod dolly wheel, and (3) a smartphone with a DJI Osmo Mobile 3 Gimbal for the smartphone). • Formal Requests for Information and Approval (1).jpg (A figure shows the formal request, in Arabic, for information and approval from Graduate Studies & Research, Faculty of Fine Arts to the transport institution: General Authority for Passengers Transport in Alexandria). • Formal Requests for Information and Approval (2).jpg (A figure shows the formal request, in Arabic, for information and approval from Graduate Studies & Research, Faculty of Fine Arts to the transport institution: Public Authority for Planning Transport Projects). • Formal Requests for Information and Approval (3).jpg (A figure shows the formal request, in Arabic, for information and approval from Graduate Studies & Research, Faculty of Fine Arts to the transport institution: National Railways Authority of Egypt). • Formal Requests for Information and Approval (4).jpg (A figure shows the formal request, in Arabic, for information and approval from Graduate Studies & Research, Faculty of Fine Arts to the transport institution: Ministry of Transport). • Formal Requests for Information and Approval (5).jpg (A figure shows the formal request, in Arabic, for information and approval from Graduate Studies & Research, Faculty of Fine Arts to the transport institution: Directorate of Housing and Utilities-Alexandria). • Translation -Formal Requests for Information and Approval (1).jpg (A figure shows the formal request, in English, for information and approval from Graduate Studies & Research, Faculty of Fine Arts to the transport institution: General Authority for Passengers Transport in Alexandria). • Translation -Formal Requests for Information and Approval (2).jpg (A figure shows the formal request, in English, for information and approval from Graduate Studies & Research, Faculty of Fine Arts to the transport institution: Public Authority for Planning Transport Projects). • Translation -Formal Requests for Information and Approval (3).jpg (A figure shows the formal request, in English, for information and approval from Graduate Studies & Research, Faculty of Fine Arts to the transport institution: National Railways Authority of Egypt). • Translation -Formal Requests for Information and Approval (4).jpg (A figure shows the formal request, in English, for information and approval from Graduate Studies & Research, Faculty of Fine Arts to the transport institution: Ministry of Transport). • Translation -Formal Requests for Information and Approval (5).jpg (A figure shows the formal request, in English, for information and approval from Graduate Studies & Research, Faculty of Fine Arts to the transport institution: Directorate of Housing and Utilities-Alexandria). Data are available under the terms of the Creative Commons Attribution 4.0 International license (CC-BY 4.0).

## References

[1] Zegras PC 1968: Sustainable urban mobility: exploring the role of the built environment. 2005 [cited 2022 Aug 17]. https://dspace.mit.edu/handle/1721.1/34170

[2] Guidelines D, Subway FOR, Development IW: TRANSIT ORIENTED COMMUNITIES DESIGN GUIDELINES FOR SUBWAY STATIONS. 2022.

[3] Michelle B Margaret G Joanne P : *Transit-Oriented Communities Design Guidelines Creating more livable places around transit in Metro Vancouver.* TransLink;2012;164. Reference Source

[4] TransLink: *Transit-Oriented Communities - A PRIMER ON KEY CONCEPTS.* TransLink;2011;1–14. Reference Source

[5] Guidelines D Subway FOR Development IW : *City of Burlington Offi cial Plan Review: Mobility Hubs Opportunities and Constraints Study.* TransLink;2011; (May):1–13. Reference Source

[6] CoMoUK: An Introduction to Mobility Hubs for Planners and Developers in Scotland. 2021. (January).

[7] Metrolinx: Green Paper #2 Mobility Hubs: Development of a Regional Transportation Plan for the Greater Toronto and Hamilton Area (for consultation). 2008;1–54. Reference Source

[8] Metrolinx: Mobility Hub Guidelines. 2011(September);1–174.

[9] National Authority for Tunnels:[cited 2022 Nov 1]. Reference Source

[10] National Authority for Tunnels:[cited 2022 Nov 1]. Reference Source

[11] National Authority for Tunnels:[cited 2022 Nov 1]. Reference Source

[12] National Authority for Tunnels:[cited 2022 Nov 1]. Reference Source

[13] Seoudy H El Menshawy A El Adawy A : Comprehensive Database for Mobility Hub Zones, 800m radius surrounding MBBT, Alexandria, Egypt.[dataset].2022; Vol.5. *Mendeley Data.* Reference Source

