# Supplementary material for: A transit map for micro-scale urban development in Alexandria, Egypt
Source: F1000Res. 2023 May 9;11:1429. Originally published 2022 Dec 5. [Version 2] doi: 10.12688/f1000research.125816.2 (PMC10189295; doi:10.12688/f1000research.125816.2)
Supplement: Peer review history

# Peer review: Reviewer response for version 2

**DOI:** 10.5256/f1000research.147365.r172454

**Published:** 2023-05-16

**Authors:** Ghada Hassan

**Affiliations:** 1 Department of Urban Design and Planning, Ain Shams University, Cairo, Egypt

All previous comments have been considered

Is the work clearly and accurately presented and does it cite the current literature?

Partly

If applicable, is the statistical analysis and its interpretation appropriate?

Not applicable

Are all the source data underlying the results available to ensure full reproducibility?

Partly

Is the study design appropriate and is the work technically sound?

Partly

Are the conclusions drawn adequately supported by the results?

Partly

Are sufficient details of methods and analysis provided to allow replication by others?

Yes

Reviewer Expertise:

Sustainable urban design, ecological landscape, urban ecology, housing

I confirm that I have read this submission and believe that I have an appropriate level of expertise to confirm that it is of an acceptable scientific standard.

# Peer review: Reviewer response for version 2

**DOI:** 10.5256/f1000research.147365.r172453

**Published:** 2023-05-11

**Authors:** Atef Ahriz

**Affiliations:** 1 Department of Architecture, University of Tebessa, Tebessa, Algeria

The author has followed all directions and made all necessary modifications

Is the work clearly and accurately presented and does it cite the current literature?

Partly

If applicable, is the statistical analysis and its interpretation appropriate?

Not applicable

Are all the source data underlying the results available to ensure full reproducibility?

Yes

Is the study design appropriate and is the work technically sound?

Yes

Are the conclusions drawn adequately supported by the results?

No

Are sufficient details of methods and analysis provided to allow replication by others?

Yes

Reviewer Expertise:

Sustainable architecture; Sustainable urban design; Energy efficiency; Sustainability; Climate; Micro-climate;  Bio climate; Urban Climatology

I confirm that I have read this submission and believe that I have an appropriate level of expertise to confirm that it is of an acceptable scientific standard.

# Response

**Published:** 2023-05-11

**Authors:** Amr El Adawy

**Affiliations:** Arab Academy for Science Technology & Maritime Transport, Egypt

Dear Dr. Atef Ahriz,

 After greeting, we would like to thank you for your report. We are glad to have your comments, which helped us upgrade our previous version of the article submission.

 Best regards and appreciation

# Peer review: Reviewer response for version 1

**DOI:** 10.5256/f1000research.138163.r168953

**Published:** 2023-04-18

**Authors:** Atef Ahriz

**Affiliations:** 1 Department of Architecture, University of Tebessa, Tebessa, Algeria

The paper entitled “A transit map for micro-scale urban development in Alexandria, Egypt” talks about a very important topic and is in line with the focus of the journal. Aiming to investigate and improve the Muharram Bek El Mowkaf El Gedid Mobility Hub (MBMH) as a crucial component of the research design employed in this study, which involved data collection, approvals, techniques, and analysis methods. The MBMH and its surrounding 800 m radius were analyzed and developed as a case study. The authors put forth a proposal for re-planning the land use of the site. Bellow the detailed review of the paper:

1. The title is suitable and clear
2. The abstract is comprehensive, well structured, and well written.
3. The paper is excellently structured.
4. Scientifically the manuscript is correct, and uses approved methods and tools to study and enhance MSTBE as a key element of the study design used in this research, depending on data collection, approvals, techniques, and analysis methods.
5. References are sufficient and recent
6. The results and discussion part are not comprehensive and detailed regarding the interest of the work. The results should be rewritten to present the results, the highlights and more discussion details.
7. The conclusion is too poor regarding the interest of the work; the conclusion must be rewritten to help the reader to understand why your research should matter to them after they have finished reading the paper.

In general, the paper is so important but it should be enhanced.

Is the work clearly and accurately presented and does it cite the current literature?

Partly

If applicable, is the statistical analysis and its interpretation appropriate?

Not applicable

Are all the source data underlying the results available to ensure full reproducibility?

Yes

Is the study design appropriate and is the work technically sound?

Yes

Are the conclusions drawn adequately supported by the results?

No

Are sufficient details of methods and analysis provided to allow replication by others?

Yes

Reviewer Expertise:

Sustainable architectureSustainable urban designEnergy efficiencySustainabilityClimate, Microclimate and BioclimateUrban Climatology

I confirm that I have read this submission and believe that I have an appropriate level of expertise to confirm that it is of an acceptable scientific standard, however I have significant reservations, as outlined above.

# Response

**Published:** 2023-04-23

**Authors:** Amr El Adawy

**Affiliations:** Arab Academy for Science Technology & Maritime Transport, Egypt

We would like to thank you, Dr. Atef Ahriz, for your comments.

 In replying to your comments, we took your comments into consideration for the new version of the article:

**In the “Results and discussion” section:**

 We rewrote it to be comprehensive and detailed regarding the interest of the work. Prior to this section, we included supplementary material in the initial sections to keep the research complete and informative, which would eventually affect the results:

- We derived the three scopes of the micro-scale transit built environment from two international case studies: the Kennedy Station Mobility Hub in Toronto, Canada, and the London King’s Cross in London, United Kingdom. We have derived lessons, from both of the case studies, about what needs to be done to move towards sustainability and completion in a micro-scale transit built environment. These lessons will be used for the research's case study development phase.
- We mentioned Collaborative Mobility UK (CoMoUK) and its Mobility Hub Accreditation to provide a set of guidelines for evaluating the quality of the case study’s mobility hub.

Out of the above points, in the “Development phase” section, we put the major points for the development of the drastic changes (changes in the land uses, heights, services, open spaces, stops, stations, streets, and urban street elements) in the current state of the whole case study area based on the demands of each mobility hub zone.

 Finally, in the "Results and discussion" section, we discussed the development in the mobility hub zones and the three scopes of the micro-scale transit built environment in detail. We ensured the distribution of the land uses was fair and acceptable under the guidance of the two international case studies. And also, we evaluated the Muharram Bek El Mowkaf El Gedid Mobility Hub using the city centre mobility hub’s CoMoUK accreditation assessment, and the mobility hub was awarded CoMoUK Gold Accreditation.

 The results were positive. In the opinion of us as authors, if the proposal is implemented as intended, it would be a starting point for a sustainable micro-scale transit built environment in Alexandria, which will affect meso-scale and ultimately macro-scale transit built environments in the long run with positive impacts all over Alexandria.

**In the "Conclusions" section:**

 We figured out that the matter of the research is that development must start from the micro-scale transit built environment to the meso-scale and then the macro-scale, as well as that people’s behaviour is always affected by the design of the micro-scale transit built environment. We applied the "Enhanced MSTBE Phases" methodology to the Muharram Bek El Mowkaf El Gedid Mobility Hub, compared land use percentages, and used the mobility hub’s CoMoUK accreditation assessment. The results were positive, which indicated our success in the methodology's application.

 Following the successful methodology’s application to the case study, we anticipated that if this method is applied to the other 22 proposed mobility hubs at MSTBEs, the influences of the MSTBEs will reach the meso- and macro-scales. Moreover, Alexandria Strategic General Urban Plans' Transportation Sector Five Goals would be achieved, which would affect the whole governorate’s urban planning and housing, urban and economic development, transportation, and the environment in the future (see Table 7).

# Peer review: Reviewer response for version 1

**DOI:** 10.5256/f1000research.138163.r162056

**Published:** 2023-02-17

**Authors:** Ghada Hassan

**Affiliations:** 1 Department of Urban Design and Planning, Ain Shams University, Cairo, Egypt

The author tried to study and enhance MSTBE as a key element of the study design used in this research, depending on data collection, approvals, techniques, and analysis methods. A case study, the analysis and development of the Muharram Bek El Mowkaf El Gedid Mobility Hub (MBMH) and the 800 m radius around it.

 The authors come up with a proposal of replanning the land use of the site.

 Many comments need to be addressed (page numbers refer to the pdf version of the article):

- p3: there is a jump to the results and suggestions that are is proven, the author imposes their opinion at the very beginning step of the research, and they might have a concrete reference to support this opinion, but they do not
- p3: a lot of repetition (sentences ad paragraphs)
- p3: the methods are mentioned in the paragraph of the study aim, while the 3 mentioned aims are too generic and are aims of project and implementation rather than aims of a research
- p5: table 1 missing reference
- p6: fig 2 missing the macroscale map to show location and its relevance, also the fig does not show bus stops as indicated in the key
-  p8-10 important that the SWOT analysis be classified according to the land use, the built form and the open spaces, must be cross referenced with the attributes cited in table 1
- Finally the conclusions are based on the result of table 5 where no clear references for its attributes
- The paper seems to be a proposal of site replanning and individual suggestions and not based on evaluation of similar examples and best practice or lesson learned.

Is the work clearly and accurately presented and does it cite the current literature?

Partly

If applicable, is the statistical analysis and its interpretation appropriate?

Not applicable

Are all the source data underlying the results available to ensure full reproducibility?

Partly

Is the study design appropriate and is the work technically sound?

Partly

Are the conclusions drawn adequately supported by the results?

Partly

Are sufficient details of methods and analysis provided to allow replication by others?

Yes

Reviewer Expertise:

Sustainable urban design, ecological landscape, urban ecology, housing

I confirm that I have read this submission and believe that I have an appropriate level of expertise to confirm that it is of an acceptable scientific standard, however I have significant reservations, as outlined above.

# Response

**Published:** 2023-04-23

**Authors:** Amr El Adawy

**Affiliations:** Arab Academy for Science Technology & Maritime Transport, Egypt

We would like to thank you, Dr. Ghada Hassan, for your comments.

 In replying to your comments, we took your comments into consideration for the new version of the article:

1. In the introduction, we did not introduce any results or suggestions, but just introduced the micro-scale transit built environment (MSTBE) as being sustainable by dividing it into three scopes, and each scope has been explained individually. As for the concrete reference to support building up sustainable MSTBE, we derived these three scopes from two international case studies in Canada and the United Kingdom (see Data collection, Methods). From these international case studies, we have derived lessons that will be useful for the research's case study development phase. In addition, we supported the scope of mobility hub with Mobility Hub Accreditation, which was developed by Collaborative Mobility UK (CoMoUK) as a tool to evaluate the quality of mobility hub in the case study.
2. We have edited the repetitive sentences in the research problem and explained that the existing MSTBEs all over Alexandria are unsustainable owing to their lack of three scopes, according to many facts including current Alexandrian TOCs, passengers, delays in receiving new transit supply system technologies, and each public transportation mode operating independently.
3. The study aims have been amended to be clearer to the reader, which are to redirect the authorities' focus from the strategic plans to be switched over to the existing MSTBEs in the cities and also to establish a method that includes phases supported by transit frameworks, strategies, solutions, and guidelines to establish an 800m radius micro-scale transit built environment. Lastly, we mentioned the objectives of the study in three points.
4. Table 1, the information in the table is extracted from our work in the case study zone.
5. Figure 2, where the circle represents the case study zone within an 800-meter radius, which equals the micro-scale within neighbourhood borders. Bus stops are clearly marked on the map (3 orange dots); they exist along the Alexandria Governorate Bus Route (magenta curve line).
6. In Table 1, we mentioned general information for the case study zone (present) as well as illustrated it in Figure 2. The information in Table 1 matches the SWOT analysis mentioned in Table 2, where we explained in detail each mobility hub zone (Primary Zone, Secondary Zone, and Tertiary Zone) and translated information of land use, built form, and open spaces in the form of figures captured in the case study. You can check this at Mendeley Data, V5, doi: 10.17632/k8hybkvsb3.5 , entitled "Comprehensive Database for Mobility Hub Zones, 800m radius surrounding MBBT, Alexandria, Egypt" (01 Primary Zone.jpg, 02 Secondary Zone.jpg, and 03 Tertiary Zone.jpg). In Addition, the upward part of Figure 5 illustrates clearly the distribution of the current land uses in the case study.
7. The "Conclusions" section was rewritten. In the “Results and discussion” section, Table 5 estimated the exact percentages of the land uses for each mobility hub zone of both the current and proposed case study states and compared them, from which we ensured that the distribution of the land uses was fair and acceptable under the guidance of the two international case studies. In the "Conclusions" section, we figured out that the matter of the research is that development must start from the micro-scale transit built environment to the meso-scale and then the macro-scale, as well as that people’s behaviour is always affected by the design of the micro-scale transit built environment. We applied the "Enhanced MSTBE Phases" methodology to the Muharram Bek El Mowkaf El Gedid Mobility Hub, compared land use percentages, and used the mobility hub’s CoMoUK accreditation assessment. The results were positive, which indicated our success in the methodology's application. Following the successful methodology’s application to the case study, we anticipated that if this method is applied to the other 22 proposed mobility hubs at MSTBEs, the influences of the MSTBEs will reach the meso- and macro-scales. Moreover, Alexandria Strategic General Urban Plans' Transportation Sector Five Goals would be achieved, which would affect the whole governorate’s urban planning and housing, urban and economic development, transportation, and the environment in the future (see Table 7).
8. Referring to the above-mentioned points, it is clear that the proposed case study was based on the evaluation of similar international case studies and lessons learned, not individual suggestions.
